# Corporate Social Responsibility Disclosure: Responding to Investors’ Criticism on Social Media

**DOI:** 10.3390/ijerph18147396

**Published:** 2021-07-11

**Authors:** Yuming Zhang, Fan Yang

**Affiliations:** School of Management, Shandong University, Jinan 250100, China; zhangym@sdu.edu.cn

**Keywords:** corporate social responsibility disclosure, social media criticism, investors, crisis management, SOEs, NSOEs, regulatory pressure

## Abstract

Companies use corporate social responsibility (CSR) disclosures to communicate their social and environmental policies, practices, and performance to stakeholders. Although the determinants and outcomes of CSR activities are well understood, we know little about how companies use CSR communication to manage a crisis. The few relevant CSR studies have focused on the pressure on corporations exerted by governments, customers, the media, or the public. Although investors have a significant influence on firm value, this stakeholder group has been neglected in research on CSR disclosure. Grounded in legitimacy theory and agency theory, this study uses a sample of Chinese public companies listed on the Shanghai Stock Exchange to investigate CSR disclosure in response to social media criticism posted by investors. The empirical findings show that investors’ social media criticism not only motivates companies to disclose their CSR activities but also increases the substantiveness of their CSR reports, demonstrating that companies’ CSR communication in response to a crisis is substantive rather than merely symbolic. We also find that the impact of social media criticism on CSR disclosure is heterogeneous. Non-state-owned enterprises, companies in regions with high levels of environmental regulations, and companies in regions with local government concern about social issues are most likely to disclose CSR information and report substantive CSR activities. We provide an in-depth analysis of corporate CSR strategies for crisis management and show that crises initiated by investors on social media provide opportunities for corporations to improve their CSR engagement.

## 1. Introduction

The disclosure of corporate social responsibility (CSR) has increased significantly in recent years, receiving growing attention from both academics and practitioners [1,2]. CSR disclosure reflects a company’s attitudes toward the interests of various stakeholders and its commitment to sustainable development [3]. Communicating CSR has significant benefits, such as improving customer loyalty and brand recognition, reducing the risk of government sanctions, lowering the cost of equity capital, and increasing competitive advantage and long-run value [4,5,6,7]. Studies have identified the specific antecedents and consequences of CSR disclosure decisions [1,4]. However, few attempts have been made to investigate companies’ CSR disclosure strategies in response to crises, especially those instigated by investors.

Crises are situations that can have devastating consequences for an organization. As a typical crisis, investors’ criticism on social media can seriously damage a company’s reputation and increase its business risks [8]. The literature has largely explored companies’ CSR disclosure decisions in response to pressure from governments, customers, the media, or the public [9]. Although investors are essential stakeholders in capital markets, investor-initiated pressure has been neglected in the research on CSR disclosure. Today, social media platforms (e.g., Facebook and Twitter) allow investors, especially small investors, to publicly voice their concerns. This may have serious consequences for firms in the firing line, as negative feedback on organizations spreads rapidly on social media [10]. Social media criticism posted by investors therefore poses a considerable threat to companies’ image and legitimacy. To minimize the associated risks, companies are likely to conduct CSR communication campaigns to manage stakeholder perceptions [11]. Focusing on crises generated by investors’ social media criticism, this study investigates whether companies respond to crises by disclosing CSR activities.

However, companies’ CSR disclosures may not fully reflect their CSR behaviors. An important issue, which remains under-explored in the literature, is the extent to which CSR disclosures are symbolic or substantive. Although many companies issue CSR reports on their social and environmental policies, practices, and performance, these disclosures may be little more than a strategic response to resolve crises rather than demonstrating substantive effort [12]. A frequently cited example is the “Beyond Petroleum” campaign by British Petroleum (BP), which makes frequent claims about its plans to invest in renewable energy to reduce global warming. However, the firm was criticized for its negative environmental impact at the Johannesburg Earth Summit [12]. Nevertheless, most studies have focused on the likelihood of companies’ disclosing CSR activities and paid little attention to the quality of the CSR communication [1,13]. To help fill this research gap, we provide in-depth insights into whether companies take substantive CSR actions or merely act symbolically in response to investors’ social media criticism.

We are also interested in assessing whether companies’ responses to social media criticism vary depending on the need for CSR communication. Social media criticism posted by investors may create varying degrees of difficulty for companies with diverse attributes, leading to heterogeneous CSR strategies. For example, Chinese state-owned enterprises (SOEs) controlled by central or local governments have considerable access to political support, even when they are heavily criticized by stakeholders [14]. As non-state-owned enterprises (NSOEs) lack government endorsement, they may be more vulnerable to criticism [13]. Therefore, the need to use communication strategies to manage societal perceptions in times of crisis is expected to be greater among NSOEs. We thus verify whether the influence of social media criticism on CSR disclosure varies between SOEs and NSOEs. In addition, regulatory pressure is a key determinant of CSR implementation [15]. When local governments place greater emphasis on environmental and social issues, CSR disclosure is considered more important. As a result, the intensity of government environmental regulation and social concern may influence companies’ decisions on CSR disclosure in response to a crisis. Therefore, we also investigate whether the effect of social media criticism on CSR disclosure differs across regions with higher and lower levels of regulatory pressure.

While CSR disclosure strategies have been widely discussed in the context of Western economies [1,4,5], relatively few studies have focused on this issue in emerging markets such as China. It is crucial to address CSR disclosure strategies in China, an increasingly important global player in CSR, for the following three reasons. First, China’s economic and manufacturing growth in the last few decades has brought significant environmental and social problems. Second, although CSR is an increasingly important item on corporate agendas in China, the quality of Chinese companies’ CSR disclosures has been questioned [14]. Third, while investors are the main participants in Chinese markets, formal legal protection for this group is weak. Social media provide a new channel for investors to influence corporate decision making [16]. Based on legitimacy theory and agency theory, we find that Chinese listed companies are more likely to disclose CSR activities when facing more criticism on social media from investors. In addition, companies facing more criticism are more likely to elaborate on the specific CSR actions taken, indicating that their responses are not merely symbolic. Furthermore, our analysis suggests that these responses are heterogeneous. The effects of social media criticism on CSR disclosure choice and substantiveness are more pronounced among NSOEs than SOEs. In addition, such criticism only has a significant effect on CSR disclosure in regions with high levels of environmental and social concern.

We contribute to the literature in several ways. First, we extend the CSR disclosure research from the perspective of crisis management. Studies have systematically explored the motivations for and outcomes of CSR activities and their disclosure [17,18]. However, CSR disclosure and its symbolic strategies in times of crisis have been underexplored. We attempt to fill this research gap by assessing both the probability and substantiveness of CSR disclosure in response to crises. Second, although investors are a powerful group with the ability to influence a company’s direction [19], studies have mainly focused on the demands of other stakeholders (e.g., governments and customers) [9,20]. We thus focus on the pressure exerted by investors through criticism on social media platforms. We use machine learning to identify such criticism and are able to correlate corporate responses with the intensity of social media criticism. Third, our findings suggest that CSR disclosure strategies in response to crises vary depending on company ownership and government intervention, providing in-depth insights into companies’ CSR communication behavior in times of crisis. Furthermore, we offer guidance for regulators on promoting CSR engagement and disclosure.

The rest of this paper is organized as follows. Section 2 introduces the institutional background. Section 3 presents the theoretical background, discusses the literature, and develops our hypotheses. Section 4 details the methodology. In Section 5, we present our empirical findings. Section 6 details further analyses of the heterogeneous impact of social media criticism. Section 7 concludes the study by describing its implications and limitations.

## 2. Institutional Background

### 2.1. CSR Disclosure in China

China is the world’s largest emerging economy. Although the country differs from the developed countries in which CSR originated [13], China is rapidly becoming a critical player in CSR. In the 1990s, when CSR initiatives were already a core business strategy in Western countries, Chinese enterprises rarely engaged in CSR [21]. To some extent, the growth of the Chinese economy at that time came at the expense of environmental considerations. Unsurprisingly, given the critical influence of the government on business and the economy in China [22], the growing emphasis on social and environmental responsibility is mainly guided by government policy. The Chinese government has begun to realize the need to balance the pursuit of economic growth with environmental protection and social development [23]. In 2003, the Scientific Outlook on Development was introduced in the Third Plenary Session of the 16th Central Committee, seeking to direct the economy toward comprehensive, coordinated, and sustainable development. Subsequently, the goal of building a harmonious society was introduced in the central government’s 11th Five-Year Plan. As a component of both China’s Scientific Outlook on Development and its plan to build a harmonious society, CSR is clearly on the national strategic agenda.

In terms of CSR disclosure, it was not until 2006 that CSR was explicitly recognized in company law in China [2]. In September 2006, the Shenzhen Stock Exchange (SZSE) issued its “Guidelines for Corporate Social Responsibility of Listed Companies.” Companies listed on the SZSE are expected to behave in a socially responsible manner and make voluntary disclosures on their performance. In 2008, the Shanghai Stock Exchange (SSE) released two sets of guidelines regarding listed companies’ social responsibility and environmental information disclosure. In the same year, the State-owned Assets Supervision and Administration Commission of the State Council (SASAC) released a document on CSR for central-government-controlled SOEs, encouraging these companies to demonstrate social and environmental responsibility and voluntarily disclose their CSR. At present, companies included in the SZSE 100 index are required to publish reports on their social responsibility. In the SSE, three types of listed companies have been required to publish CSR reports since 2008, namely, companies listed in the Corporate Governance Index, companies that list shares overseas, and companies in the financial sector [13]. Other companies listed on both stock exchanges are encouraged to make CSR disclosures on a voluntary basis. In the short history of CSR disclosure in China, government promotion has rendered CSR disclosure an increasingly important component of business, attracting the attention of various stakeholders.

**Figure 1:**
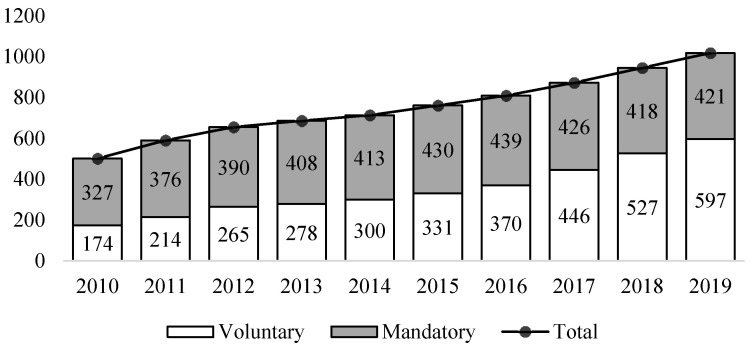
The number of CSR disclosure reports issued by Chinese listed firms from 2010 to 2019.

Figure 1 presents the number of Chinese A-share listed companies that disclose CSR information, according to data from the China Security Markets and Accounting Research (CSMAR) database. Due to severe data limitations before 2010, we analyze only data from 2010. As shown, Chinese listed companies gradually increased their CSR disclosure practices from 2010 to 2019. In 2017, the number of voluntary CSR disclosures exceeded the number of mandatory disclosures for the first time. From that year onward, a stable increase in the proportion of voluntary CSR disclosure can be observed. In the latest available year (2019), 597 of the 1018 CSR disclosures issued were voluntary, three times greater than in the starting year, 2010. These numbers indicate that Chinese listed companies are gradually recognizing the value of voluntary disclosure. However, there is still a significant gap between China and Western countries in terms of the level of CSR disclosure. To supplement the research on emerging markets, it is crucial to assess Chinese firms’ social responsibility initiatives and disclosure strategies.

### 2.2. The Use of Social Media in Capital Markets and Potential Crises

We focus on small investors as company stakeholders. Whereas developed capital markets are dominated by institutional investors, small investors are the main participants in emerging markets. In China, small investors account for 99.77% of stock market participants (according to Chinese statistics, the number of investors at the end of January 2021 was 179,869,200, of which natural persons numbered 179,448,300 (data source: China Securities Depository and Clearing Corporation Limited, CSDC)) and thus play an important role in corporate governance. How best to respond to the demands and manage the perceptions of this group is a vital question for companies in emerging markets. In the last two decades, the managers of listed companies have sought to interact with large institutional stakeholders through road shows, conference calls, and site visits [10]. However, they have neglected small investors, as the formal legal protections for such investors are relatively weak in emerging economies [16]. Therefore, this group has had to seek alternative channels, such as new forms of media [24,25], to influence company decisions [26].

Along with the growing use of information technology in capital markets, social media platforms have become an increasingly important tool for firm–investor communication [10]. In 2013, the U.S. Securities and Exchange Commission (SEC) issued guidelines that explicitly approved companies’ use of social media platforms to announce information (see https://www.sec.gov/news/press-release/2013-2013-51htm#.VM9R3u8rfc, accessed on 10 July 2021)). In the same year, the SSE built a formal social media platform known as E-interactive (see http://sns.sseinfo.com, accessed on 10 July 2021)), aiming to encourage two-way interaction between listed companies and investors. Since then, Chinese market participants, especially small investors, have had a direct line with which to communicate with managers. Given their interactive nature, social media have changed not only the process of companies’ information dissemination but also corporate governance [27]. Social media provide investors with the opportunity to process and disseminate information, potentially challenging top managers in a public forum. However, social media are a double-edged sword, as they can trigger a crisis by broadcasting damaging news to a wider audience, thereby accelerating the spread of negative sentiments. Due to the unpredictability of the fallout from social media criticism, firms have lost a certain amount of control over their information environments [28]. As a result, if an investor publicly denounces a company’s actions on social media, the company will treat the event as a crisis, irrespective of whether the accusation is true. Social media criticism sometimes goes viral, with devastating consequences for corporate reputation. It is therefore vital for companies to respond adequately to social media criticism.

## 3. Theoretical Background and Hypothesis Development

### 3.1. Theoretical Background

Although many CSR studies have been conducted, no single theory frames the issue of CSR in response to social media criticism by investors. While some studies have discussed the mechanisms of companies’ CSR engagement and disclosure using either institutional theory, legitimacy theory, stakeholder theory, or agency theory [29,30], our study has a multi-theoretical framework: we use legitimacy theory and agency theory to analyze CSR communication strategies in response to crises.

Legitimacy theory, widely used in CSR studies [31], stipulates that in a social system, there is a social contract between corporations and society. A company needs to seek and maintain legitimacy by complying with social values and must operate conforming to societal expectations [32]. Legitimacy is critical capital that provides organizations with access to resources allocated by stakeholders, brings competitive advantages, and enhances their long-run value [4,7]. Failing to respond to public expectations regarding legitimate conduct usually results in sanctions against the offending business. In this study, we focus on criticism posted on social media by investors that posed a threat to organizational legitimacy and plunges companies into crisis. According to legitimacy theory, an organization can rebuild its reputation by communicating its commitment to society rather than appearing to take actions that are motivated by self-interest [33]. As a result, legitimacy theory provides an appropriate framework to explain companies’ CSR disclosure behavior in times of crisis.

Agency theory has also been widely used in the literature on CSR disclosure, as it links various corporate governance mechanisms to CSR decision making in corporations [18,29]. According to agency theory, effective mechanisms have an impact on managerial CSR decisions [34]. For example, Majeed et al. [18] found that institutional investors, independent directors, foreign nationals, and female directors were most likely to support CSR-related activities, and that there were significant associations between these identity markers and the extent of CSR reporting [35]. As a significant source of crisis for a company, investor-driven social media pressure also plays a governance role: investors’ posts serve to monitor decisions in a corporation by airing public opinion, as indicated by Ang et al. [16]. Therefore, this theory provides insights into agency issues regarding CSR disclosure and how the fallout from crisis, including crises caused by social media criticism, can be overcome through effective governance mechanisms.

### 3.2. Literature Review

Corporate disclosures are a prerequisite for the efficient allocation of resources in capital markets and for building stakeholder confidence [36]. Corporations recognize that disclosing CSR activities is a key way of cultivating legitimacy, as it enables them to demonstrate their efforts to become more socially responsible and accountable [31]. Furthermore, CSR is an important mechanism for building corporate reputation and prestige [3,37]. Studies have posited that CSR disclosure helps companies to attract institutional investors [38,39], enhances companies’ relationships with stakeholders and the government [11], reduces the cost of equity capital [5,17], enhances companies’ reputation, gives companies a competitive advantage over their rivals [40], and enhances companies’ long-term performance and value [7]. With widespread recognition of the importance of CSR disclosure in business, a large and growing body of literature has examined the determinants that drive CSR disclosure. From the perspective of legitimacy, CSR reporting serves as a communication strategy and legitimizing tool to influence public perceptions of companies [31]. Given the public’s tremendous influence on businesses, companies have an incentive to provide CSR disclosures when they face pressure from investors, governments, customers, media representatives, and mimetic stakeholders [9,41]. Furthermore, strategic decisions such as CSR disclosure is a function of corporate governance mechanisms [29]. A number of studies have documented a significant relationship between CSR disclosure and firm ownership, board structure, internal control, and top management team composition [1,18,29]. Amid the extensive research on CSR disclosures and various crises, there has been much exploration of how companies conduct CSR activities to manage crises in the face of pressure from customers, governments, the media and the public. However, there is minimal empirical evidence of strategic CSR activities in response to crises instigated by investors, particularly investors’ criticism on social networks.

As an important form of non-financial disclosure, CSR disclosures are expected to contain comprehensive and in-depth information regarding companies’ policies, practices, and performance in the social and environmental domains [42]. Unlike financial disclosure, which is mandatory and verifiable, CSR disclosure is unregulated in China; it is mainly published on a voluntary basis and lacks a widely enforced reporting framework. As a result, CSR disclosure lacks external audit and regulatory oversight [42]. Whereas financial reports frequently use quantifiable data to illustrate a company’s financial performance, CSR disclosure primarily provides qualitative textual information related to a company’s sustainable development practices in areas such as production safety, environmental protection, and community welfare [17]. Due to the descriptive and unverifiable nature of CSR disclosure, disclosure quality plays a prominent role in determining the effectiveness of CSR communication and in managing stakeholders’ perceptions of companies’ CSR activities [1]. Du and Yu [42] argued that CSR reports released by companies should convey useful information to market participants and improve information transparency. Lee et al. [13] posited that state subsidies influence Chinese firms’ decisions on whether to disclose extensive CSR information, citing political cost considerations. Nevertheless, a few studies have documented that companies often comply with stakeholder demands by strategically supplying CSR disclosures, although of low quality and containing limited information. For example, Lyon and Maxwell [12] criticized companies for using a “greenwashing” (greenwashing refers to the organizational practice of strategically disclosing environmental information but failing to fully demonstrate the organization’s environmental impact to deflect attention from its environmentally unfriendly or less savory activities) communication strategy to publicize environmental actions that they have not actually taken and to mask their actual environmental practices, thereby misleading the public. Taking an institutional perspective, Marquis and Qian [14] also showed that companies in emerging markets often respond only symbolically to government requests for CSR reporting. Therefore, there is so far limited and inconclusive evidence on whether companies provide CSR disclosure in a substantive way when under pressure or facing threats.

### 3.3. Hypothesis Development

#### 3.3.1. CSR Disclosure Decision in Response to Social Media Criticism

CSR decisions in corporations are made under social pressure in social environments. Based on legitimacy theory, companies face increasing pressure and criticism from stakeholders and are thus increasingly expected to offer voluntary CSR disclosure to address increased threats to their organizational legitimacy [31]. In contrast to traditional, often one-way communication methods such as press releases and conference calls, social media create richer channels for the dissemination of valuable information among market participants [43]. Although social media platforms provide some advantages for companies, they can also create challenges to the management of the flow of information; due to the platforms’ interactive qualities, companies have little control over what users post. As a result, companies have become vulnerable to threats to their organizational prestige [10]. When investors use social media to publicly criticize a company, their negative comments are widely disseminated to other users and impact perceptions of the company. Social media criticism posted by investors negatively affects companies’ reputation and thus their performance and stock prices. Such posts also increase the probability of companies’ being investigated by regulators, which may expose their illegal or unethical activities, further impairing organizational legitimacy [32]. Furthermore, the loss of legitimacy may lead to regulatory penalties, damage business networks, and limit companies’ access to necessary resources. To minimize these adverse effects, companies strive to present themselves as good corporate citizens to stakeholders [3]. The most efficient way for companies to improve their reputation is to implement legitimization strategies, such as voluntary CSR disclosure to declare their CSR initiatives, to strengthen the trust of investors and other stakeholders. According to Deegan [44] and Cho and Patten [31], companies seeking to create reputational capital can use communication strategies such as social and environmental disclosures. Similarly, by portraying companies in a positive light and influencing societal perceptions accordingly, the disclosure of CSR activities can serve as a crisis management tool to foster legitimacy in response to crises brought by social media criticism.

From the perspective of corporate governance, we posit that social media criticism predicts the decision to disclose CSR information given its monitoring role. Although numerous CSR studies carried out in Western contexts have emphasized the managerial over investment tendency in CSR, there is a comparative lack of enthusiasm for CSR engagement among companies in emerging countries. Due to the separation of ownership and control in corporations [45], those in managerial positions are often the owner’s agents. These agents do not bear all the consequences of CSR for a firm’s long-term value [34]. Instead, managerial CSR decisions become myopic because substantial financial resources are needed to support these activities, which often give rise to short-run performance volatility [46]. According to agency theory, effective governance mechanisms can play a monitoring role in mitigating agency issues affecting managerial decisions [47]. For example, a variety of factors, such as institutional ownership, board independence and media coverage, can have positive impacts on CSR decisions [29,35]. Zhou et al. [16] found that attention garnered through investors’ social media posts encouraged companies to take timely corrective action to redress violations, confirming that public criticism on social media is an effective mechanism of corporate governance. Based on the aforementioned argument, we believe that social media criticism posted by investors can play an important role in motivating managers to make value-added decisions by behaving more ethically, thereby resulting in voluntary CSR disclosure.

CSR disclosure serves as a legitimizing tool used by companies to communicate with stakeholders, as it helps mitigate the effects of potential losses due to increasing criticism. Moreover, social media criticism exposes companies’ decisions to greater monitoring, which encourages CSR involvement and disclosure. As such, we propose the following hypothesis to guide our empirical investigation:

**Hypothesis 1** **(H1).** *Companies subject to high levels of social media criticism by investors are more likely to disclose voluntary CSR information.*

#### 3.3.2. CSR Disclosure Substantiveness in Response to Social Media Criticism

Arguing that a company’s disclosure strategies are not usually scrutinized by stakeholders, some research has failed to consider the substantiveness of information disclosure [48]. However, with increasing attention devoted to CSR practices, CSR reports that contain inaccurate or overly positive descriptions carry significant risks for companies, especially those subject to pressure from critical stakeholders. Research on CSR disclosure strategy has also suggested that deliberately failing to provide substantive CSR disclosures has adverse consequences for companies, as stakeholders (e.g., the public, media, government) might expose the companies’ lies and punish them accordingly [14]. The Chinese newspaper *Southern Weekly* releases an annual “greenwashing list” detailing unethical behavior by Chinese corporations, particularly greenwashing. In 2017, *Southern Weekly*’s greenwashing list included FAW Group, a company that publicized its “green factory and green industrial chain” program in one of its CSR reports. Although seemingly an environmentally responsible program, the report ignored FAW Group’s questionable practices, including poor pollutant treatment and excessive emissions of harmful waste gas. The public reacted angrily to the company’s dishonesty. As a result, FAW Group was criticized and fined by the government. The FAW Group incident demonstrates the ways in which corporate CSR actions are monitored by stakeholders: symbolic (non-substantive) CSR gestures not only increase the risk of disrepute but also have negative economic outcomes. Investors’ social media criticism places companies under the watchful eye of sophisticated stakeholders such as analysts, auditors, the government, and the media. Therefore, in times of crisis, failing to provide substantive CSR disclosures is likely to exacerbate the spread of negative publicity via social media, increasing the threat to organizational legitimacy. To rebuild the confidence of stakeholders and reduce business risk, companies are likely to cultivate a positive image by engaging in substantive CSR disclosure in response to social media criticism.

In addition, when companies publicize ethical actions that they have not actually taken in CSR reports, they engage in opportunistic behavior, which arises from agency problems. Given the monitoring role of social media [16,27], we expect social media criticism posted by investors to be closely associated with the substantiveness of CSR disclosures. Through substantive CSR actions, companies are able to make substantive progress, including the adoption of clean production methods, advancement of technological innovation, and increased research and development for green products. Such activities are conducive to reducing production costs, improving customer satisfaction, and increasing competitive advantage. On the contrary, responding merely symbolically to stakeholders’ demands is not beneficial to a company’s long-term value and may have devastating consequences if deception is discovered. Social media criticism posted by investors has an important influence on the ways in which a company operates. The negative publicity brought by stakeholders’ criticism has a deterrent effect, encouraging companies to act in socially and environmentally responsible ways. This is in line with the argument of Campbell [30], and Marquis and Qian [14] that companies’ reporting of symbolic or substantive CSR activities depends on the extent of external scrutiny or monitoring. It can be assumed that in crisis situations, in which companies are likely to be monitored and criticized by investors, companies are more likely to provide substantive CSR disclosures. We therefore hypothesize as follows:

**Hypothesis 2** **(H2).** *Companies facing more social media criticism from investors are likely to engage in more substantive CSR disclosure.*

## 4. Methodology

### 4.1. Sample and Data

Our data come from several credible sources. Specifically, our CSR disclosure choice data come from the CSMAR database and our CSR disclosure substantiveness data come from an independent CSR ratings agency, Rankins CSR Ratings (RKS). The CSMAR database provides information on Chinese companies’ CSR activities as disclosed in their CSR reports or annual reports. RKS provides highly credible information on the substantiveness of CSR reporting, in line with the Global Reporting Initiative (3.0). Studies have also shown it to be a credible source of data on the substantiveness of CSR reporting [13]. To collect data on social media criticism, we use Python to crawl all comments posted by investors on each listed company on the E-interactive platform, which is discussed in Section 2. This platform is especially useful for investigating our hypotheses for two reasons. First, as it was created by a stock exchange, the E-interactive platform differs significantly from other online channels (e.g., Facebook, Twitter, Weibo): it serves participants in the stock market, especially investors and companies, and thus to a great extent avoids interference from other agents, such as consumers. Using this platform can help researchers observe investors’ comments on a company, providing a useful method of measuring the extent of a crisis. Second, the platform features a specialized Q&A section and communication on the E-interactive platform is initiated by investors rather than companies. According to the rules of the SSE, if companies receive questions or criticism from investors, they must reply or take relevant actions in a timely manner. Therefore, investors’ concerns on this platform are expected to have a more significant influence on corporate decision making than posts on other social media. The information on institutional ownership information comes from the Wind Financial Database (Wind). All other relevant data are obtained from the CSMAR database.

To investigate CSR disclosure choice, we base our sample selection on all Chinese companies listed on the SSE from 2013 to 2019. We use 2013 as the start date because the SSE developed the E-interactive platform in that year. Prior to this year, there was no formal social media platform for small investors to publicly interact with the management of the firms listed on the SSE. We restrict our sample to non-financial companies. Table 1 illustrates the details of our sample. Panel A presents our sample selection process for testing reporting choice. Of the 8703 observations for 2013 to 2019, we eliminate 2253 observations that feature mandatory CSR reports, as we consider only voluntary CSR reporting choice. We then eliminate 451 observations with missing values for the variables used in the subsequent empirical tests. This selection process results in a final sample of 5999 firm-year observations. Panel B provides the yearly distribution of the observations and demonstrates that the percentage of Chinese firms that voluntarily disclosed CSR information increased gradually across the sample years. For example, the years with the smallest and largest proportions of firms that voluntarily disclosed CSR information are 2013 and 2019, at 14.216% and 23.931%, respectively.

To investigate the substantiveness of firms’ CSR reporting, we use data for the period 2013 to 2017, as the most recent open dataset on firms’ CSR reporting is only available from RKS up to 2017. Following Lee et al. [13], we incorporate companies that are required to issue CSR reports into our sample to provide comprehensive evidence of the substance of CSR disclosure and maximize the sample size. Panel C summarizes the sample selection process. The sample is limited to companies with CSR reports rated by RKS. Due to missing values for CSR reporting rating scores and other variables, the final sample for the test is reduced to 2099 firm-year observations, slightly less than the initial sample of 2249. Panel D provides the yearly distribution of the sample. The average rating score over the whole sample period does not exceed 50 out of 100, indicating that the substantiveness of Chinese listed companies’ CSR disclosure is poor.

### 4.2. Variable Definitions

#### 4.2.1. CSR Disclosure

We focus not only on CSR disclosure choice but also on the substance of CSR activities reported. Thus, we consider two dependent variables. First, we use an indicator variable, CSR, to designate a company’s CSR disclosure choice. We assign CSR a value of 1 if a company voluntarily issued a standalone CSR report or disclosed CSR-related information in its annual report in a given year; otherwise, we assign CSR a value of 0.

Second, we assess the extent to which CSR disclosure reflects substantive CSR activities. Following Marquis and Qian [14], we use the extent to which CSR disclosure is substantive (RKS) as another dependent variable, which is measured as an overall rating score of CSR reporting from an entirely independent CSR rating agency, RKS, which has been widely used by researchers [14,18]. Specifically, on the basis of data gathered from CSR reports as well as other communications such as websites and press releases, each firm’s CSR reporting is evaluated in four dimensions. The first is an overall evaluation based on a firm’s CSR strategy, the extent of stakeholders’ participation in CSR activities, the comparability of CSR disclosure across time, the innovativeness of CSR activities, and the extent of external auditing. The second is a content evaluation based on the extent of the leadership and organizational system in place for implementing CSR activities. The third is a technical evaluation based on the transparency, regularity, and availability of CSR information. The fourth is an industrial evaluation that focuses on CSR activities associated with industry-specific factors. The overall rating score for CSR disclosure substantiveness ranges from 0 to 100. As such, we take the natural logarithm of the rating score as the dependent variable.

#### 4.2.2. Social Media Criticism Posted by Investors

We focus on how companies strategically manage specific crises brought by social media criticism through CSR communication. Unlike past studies [9], we focus exclusively on companies criticized by investors on social media platforms to understand the unique characteristics of this type of criticism and its effects on organizations. We identify the magnitude of a crisis according to the volume of criticism posted over time.

Moreover, we identify the criticisms themselves. On the E-interactive platform, individual investors can publish positive as well as negative comments on a company. A growing body of capital market and information disclosure research has conducted textual analyses to examine the tone and sentiment of corporate annual reports, press releases, conference calls, and media reports [49,50,51]. In this study, we also distinguish between positive and negative posts based on investors’ tone, using textual analysis methods. There are two feasible approaches to conducting textual analysis: the dictionary-based approach and the machine learning method. The former approach uses an algorithm in which a computer program reads the text and classifies words (or phrases) into different categories (i.e., positive versus negative) based on a dictionary (word list). For instance, a general-purpose dictionary such as the Harvard General Inquirer is often used to perform textual analysis. Loughran and McDonald [52] developed a financial emotion word list (LM list) that captures positive and negative words in the accounting and finance contexts, which has been widely used in capital market research [42,49].

The alternative method, machine learning, can automatically infer the content of a text and classify documents with subjective opinions based on big data and deep learning techniques. In this study, we use machine learning instead of the dictionary-based method for several reasons. First, very few word lists are appropriate for use in research on Chinese capital markets. Li [53] argued that dictionary-based approaches are not ideal for finance research, even though this method has been used extensively in the fields of Western languages and culture. In the Chinese context, researchers have usually translated the English words in the LM dictionary into Chinese and measured tone by calculating negative/positive word frequencies based on the translated word lists [54]. However, this process introduces considerable bias and renders the method inapplicable to the Chinese context, as there are significant differences in linguistic elements such as word meanings, expressions, sentence structure, and cultural elements between the Chinese and English languages. Second, dictionary-based approaches do not take sentence context into consideration. For instance, if an investor is talking about revenue, “increase” should be treated as a positive word; however, the word is likely to have a negative meaning in the context of cost [53]. Fortunately, the machine learning approach can address these problems.

We use Senta, an open-source sentiment classification system established by Baidu that uses the Bidirectional Long Short-Term Memory (Bi-LSTM) model, to perform our sentiment analysis. The LSTM model is a type of recurrent neural network model that overcomes the vanishing gradient problem. The model learns to store only relevant content based on training [55]. The Bi-LSTM model has been pre-trained with large corpora and is intended for widespread use in various domains, such as e-commerce, tourism, media, finance, and law. The model accepts the input of a given sentence and automatically gives a positive sentiment score (ranging from 0 to 1) and a negative sentiment score (ranging from 0 to 1) for specific texts. The effectiveness of the Bi-LSTM model is high; a recent study found that the model’s accuracy with a test set exceeded 85% [56]. According to the metrics of sentiment analysis, comments on the E-interactive platform that receive scores higher than 0.5 are identified as negative and therefore defined as “criticism”. We calculate the natural logarithm of 1 plus the total number of criticisms posted by investors about a company on the E-interactive platform in a given year to proxy for social media criticism (CRITICISM).

### 4.3. Model Specification

To test H1, we estimate the probability of a company’s disclosing CSR information. As a dummy variable is used as the dependent variable, a probit regression model is applied as follows:

(1) $$\begin{array}{cc} \mathrm{Pr}\left({\mathrm{CSR}}_{i,t}\right) & =\alpha_{0}+\alpha_{1}{\mathrm{CRITICISM}}_{i,t}+\alpha_{2}{\mathrm{SIZE}}_{i,t}+\alpha_{3}{\mathrm{LEV}}_{i,t}+\alpha_{4}{\mathrm{ROA}}_{i,t}+\alpha_{5}{\mathrm{TOBINQ}}_{i,t}\\ & +\alpha_{6}{\mathrm{INSH}}_{i,t}+\alpha_{7}{\mathrm{FSH}}_{i,t}+\alpha_{8}{\mathrm{OUTDIR}}_{i,t}+\alpha_{9}{\mathrm{BOARD}}_{i,t}+\alpha_{10}{\mathrm{SOE}}_{i,t}\\ & +\mathrm{Industry}+\mathrm{Year}+\varepsilon_{i,t} \end{array}$$

To test H2, we use ordinary least squares (OLS) regression to examine the relationship between criticism and the substance of CSR disclosure as follows:

(2) $$\begin{array}{cc} {\mathrm{RKS}}_{i,t} & =\beta_{0}+\beta_{1}{\mathrm{CRITICISM}}_{i,t}+\beta_{2}{\mathrm{SIZE}}_{i,t}+\beta_{3}{\mathrm{LEV}}_{i,t}+\beta_{4}{\mathrm{ROA}}_{i,t}+\beta_{5}{\mathrm{TOBINQ}}_{i,t}\\ & +\beta_{6}{\mathrm{INSH}}_{i,t}+\beta_{7}{\mathrm{FSH}}_{i,t}+\beta_{8}{\mathrm{OUTDIR}}_{i,t}+\beta_{9}{\mathrm{BOARD}}_{i,t}+\beta_{10}{\mathrm{SOE}}_{i,t}\\ & +\mathrm{Industry}+\mathrm{Year}+\mu_{i,t} \end{array}$$

where i indexes firm and t indexes time. The dependent variable of CSR disclosure choice, CSR, is a dummy variable that equals 1 if firm i voluntarily issues a standalone CSR report or discloses CSR information in its annual report in year t, and 0 otherwise. RKS is the natural logarithm of the overall rating score of CSR disclosure substantiveness for a firm in year t, obtained from RKS. The independent variable, CRITICISM, is the natural logarithm of 1 plus the volume of criticism (negative posts) posted by investors on firm i via the E-interactive platform in year t. We estimate Equations (1) and (2) with a focus on α_1_ and β_1_, the coefficients for CRITICISM, which are expected to be significantly positive if companies tend to disclose CSR information and issue substantive CSR disclosures in a severe crisis arising from social media criticism.

To eliminate potential confounding effects, we introduce a series of control variables that may impact CSR disclosure choice or quality to the regression model. Following Lee et al. [13], we expect larger firms to face more public pressure, to have more financial resources to engage in CSR activities, and therefore to provide more substantive disclosures. As a result, we include SIZE, which is computed as the natural logarithm of the book value of assets at the end of year t. Firm leverage increases business risk, which in turn requires companies to make CSR disclosures [57]. We measure leverage (LEV) using the ratio of total debt divided by total assets at the end of year t. Following the argument of Li et al. [46] that well-performing companies may have greater incentives to disclose their CSR activities to stakeholders, return on assets (ROA) (the ratio of net income divided by total assets in year t) should be controlled. Higher-growth firms may either have greater resource constraints that inhibit their ability to issue good CSR reports [13] or have more motivation to voluntarily initiate non-financial disclosure to reduce the cost of capital [17]. Therefore, there is no way to predict with certainty a company’s growth opportunity and CSR disclosure. We therefore measure growth opportunity by Tobin’s Q (TOBINQ), which is computed as the ratio of the market value to the book value of assets in year t. Majeed et al. [18] documented a variety of elements related to corporate governance that have significant effects on companies’ CSR engagement and disclosure decisions. To capture these effects, we include several governance-related control variables: institutional ownership (INSH), the percentage of stock holdings by institutional investors at the end of year t; ownership concentration (FSH), the percentage of stock holdings by the first largest shareholder at the end of year t; independent directors (OUTDIR), the proportion of independent directors on the board in year t; and board size (BOARD), proxied by the natural logarithm of the number of directors on the board in year t. In the Chinese context, SOEs are encouraged by the state to be model corporate citizens, and are therefore more likely than NSOEs to engage in CSR activities and demonstrate their CSR initiatives through substantive disclosure [41]. We therefore include SOE in the regression model to specify whether a firm is an SOE. Finally, dummy variables representing China Securities Regulatory Commission (CSRC) two-digit industry membership and year fixed effects are included in all of the regressions. The specifications of the variables used in our empirical analyses are presented in Table 2.

## 5. Empirical Findings

### 5.1. Descriptive Statistics

Table 3 presents descriptive statistics for the variables used in our analyses. To mitigate the influence of outliers, all of the continuous variables are winsorized at the 1st and 99th percentiles. The mean value of CSR is 0.186, with a standard deviation of 0.389, which indicates that Chinese companies that voluntarily disclosure CSR information account for only 18.6% of the observations in our sample. The average CSR disclosure substantiveness is 3.711 and the corresponding original value is 40.895, with minimum and maximum values of 21.200 and 82.517, respectively. This means that, on average, Chinese companies’ CSR reports contain only limited information on specific CSR activities, although there are big differences between companies in the sample. Our results show that CRITICISM has an average value of 2.816 and an original value of 15.710. The standard deviation for CRITICISM is 1.175 and the minimum and maximum values for CRITICISM are 0.000 and 5.298, respectively. These results imply that, on average, a firm in our sample was criticized by investors 16 times on the E-interactive platform each year, with significant variation in the extent of crisis across companies. In particular, the most heavily criticized firm received approximately 199 negative posts in the focal period, while a few firms did not receive any criticism on the platform. The average value of institutional ownership (INSH) is 0.386, suggesting that the average ratio of institutional ownership of our sample is 38.6%. Regarding the other control variables, the average firm has a SIZE value of 22.185, leverage of 0.454, return on assets of 0.033, Tobin’s Q of 2.295, largest shareholder ownership of 0.365, and board size of 2.134. On average, independent directors make up 37.3% of a firm’s board members. SOEs account for 42.9% of the observations. Overall, the statistical distributions of the above variables are consistent with previous studies [13].

### 5.2. Pearson Correlation Analysis

Table 4 shows the Pearson correlation matrix for the main variables. The correlation between CSR and CRITICISM is 0.062, significant at the 1% level. The preliminary correlation results indicate a positive relationship between social media criticism and the possibility of CSR disclosure, which lends initial support for H1. The correlation between RKS and CRITICISM is 0.025, but it is not statistically significant. The two dependent variables have positive significant correlations with SIZE, LEV, INSH, FSH, and BOARD. The correlations between CSR, RKS, and SOE are also significantly positive, in line with our expectation that state-owned enterprises are more likely to disclose CSR activities and issue high-quality CSR reports. Moreover, the two CSR variables have positive relationships with TOBINQ. Finally, as the maximum pairwise correlation between OUTDIR and BOARD is −0.560, below the maximum threshold of 0.7 suggested by Lind et al. [58], multicollinearity is not a serious concern in this study. Although most of the results are consistent with our expectations, Pearson correlation analysis does not simultaneously control for other variables. We examine the relationships more rigorously in subsequent analyses.

### 5.3. Test of H1: CSR Disclosure Choice

Table 5 presents probit regression estimates of the probability of a company’s disclosing CSR information. Column (1) introduces CSR as the dependent variable and controls for other determinants of CSR disclosure identified by previous studies as well as industry and year fixed effects. It shows that the coefficient of CRITICISM is positive, as predicted, and significant at the 1% level (coefficient = 0.066, z-stat = 3.788). The empirical results suggest that being criticized more severely by investors on social media platforms results in companies’ making voluntary CSR disclosures, supporting H1. Based on the marginal effect reported in Column (2), a 1% increase in CRITICISM increases the likelihood of companies’ issuing CSR disclosure by 0.016%. Our findings indicate that in response to an increasing crisis brought by social media criticism posted by investors, listed companies are more likely to disclose their CSR activities voluntarily. The findings are in line with our argument that listed companies anticipate the negative consequences of a crisis when faced with increasing investor criticism on social media platforms. To mitigate the negative effects of this criticism, companies are expected to conduct crisis management and rebuild legitimacy using CSR disclosure to communicate their social and environmental initiatives to stakeholders. In addition, as Zhou et al. [16] and Ang et al. [27] documented using the principal–agent framework, social media criticism by investors serves as an effective mechanism of corporate governance, as it forces managers to make value-added decisions, including voluntary CSR disclosure [18].

The signs of the coefficients on most of the control variables are as expected, apart from LEV and ROA. For example, the coefficient of SIZE is significantly positive in the regression, as expected, suggesting that large firms have greater incentives to voluntarily disclose their CSR actions. INSH also has a positive coefficient, consistent with the argument that institutional investors place a high value on social achievements and reputation when investing in firms, and companies with higher institutional ownership are more likely to voluntarily issue CSR reporting [18]. The variable BOARD is positively related to CSR disclosure choice, implying that board size has a positive influence on companies’ voluntary CSR disclosure. Moreover, the coefficients on SOE are significantly positive as expected, indicating that Chinese SOEs engage more actively in CSR activities than non-SOEs and have greater incentives to make CSR disclosure decisions. Firm leverage (LEV) and return on assets (ROA) also have significant coefficients, but their relationships with the probability of CSR disclosure are negative in this case, indicating that poor-performing and low-leverage Chinese firms are more likely to issue CSR reports or disclose CSR information in their annual reports. The results are consistent with the findings of other relevant studies in the Chinese context [1,13].

### 5.4. Test of H2: CSR Disclosure Substantiveness

Above, we establish that companies tend to disclose CSR information to manage stakeholders’ perceptions. To test H2, we investigate the substance of the CSR disclosures released by conducting further analyses. Table 6 reports the results of the OLS regression model of CSR reporting substantiveness in response to social media criticism, controlling for other factors that are also related to the dependent variables. We note that the coefficient of CRITICISM is positive and statistically significant at the 5% level (coefficient = 0.011, t-stat = 2.389), lending support for our second hypothesis, H2. Therefore, we can conclude that increasing social media criticism posted by investors pushes companies to issue CSR reports with more substantive details that reflect what they have actually done to address the issues raised in the criticism. Conversely, if companies only strategically issue symbolic reports that are uninformative, investors and stakeholders are likely to become increasingly angry and resentful, especially in times of crisis. Companies recognize that providing substantive details in CSR reports is a useful way of managing stakeholders’ perceptions and regaining legitimacy: effective communication regarding company initiatives through CSR actions provide a transparent means of pacifying investors and other stakeholders in times of crisis. As a result, companies are more likely to publish greater substantive CSR reports to enhance their reputation and legitimacy in response to social media criticism posted by investors.

Regarding the control variables, most of the results are as expected. Specifically, the significantly positive coefficients of SIZE and BOARD support the findings of Cabeza-García et al. [1] that firm size and board size have positive effects on the substance of CSR reporting. The growth opportunity (TOBINQ) of firms is positively related to CSR disclosure substantiveness, probably because Chinese firms with greater growth opportunities are motivated to initiate detailed CSR disclosure to acquire financial resources for growth from stakeholders. Consistent with Majeed et al. [18], we still find institutional ownership (INSH) to be positively related to the substantiveness of CSR reporting. SOE has a significant coefficient of determination, which is consistent with the assumption stated in the previous section that Chinese SOEs are encouraged to conduct activities that are beneficial to society and the environment, and thus are more likely to engage in more substantial CSR disclosures than NSOEs. Moreover, while the variable FSH is positively correlated with CSR disclosure substantiveness, this is not statistically significant, and there is also no evidence pointing to a relationship between LEV, ROA, OUTDIR, and the dependent variable.

### 5.5. Robustness Checks

#### 5.5.1. Alternative Proxy for CSR Disclosure Substantiveness

To ensure the reliability of our findings, we perform a robustness test by replicating the main tests with an alternative measure of CSR disclosure substantiveness. In addition to the ratings score used in our main tests, which is a continuous variable, RKS ranks companies each year according to their overall score for CSR reporting substantiveness on 17 levels, ranging from CC to AA+. AA+ is reserved for the highest-quality CSR reports, which include the greatest amount of substantive detail, and CC is reserved for the lowest-quality CSR reports, which are symbolic and/or uninformative. Following Lee et al. [13], we conduct robustness tests with this alternative dependent variable (RKS_rank), which ranges from 1 to 17. A Poisson regression model is therefore employed, as the variable RKS_rank is count based. When RKS_rank is taken as the dependent variable, the results reported in Table 7 still show a positive and significant relation between social media criticism posted by investors and CSR disclosure substantiveness at the 5% level (coefficient = 0.017, z-stat = 2.368). Thereafter, most of the control variables have signs in line with our expectations. These findings suggest that the main hypothesis is still valid, even after employing alternative methods to measure the degree of substantiveness of CSR activities.

#### 5.5.2. Condensed Sample

Our sample contains a small number of companies not subject to any social media criticism posted by individual investors. In the primary analysis above, the variable CRITICISM takes a value of 0, as these observations were not actually threatened by a specific crisis in our sample period. To further enhance the robustness of our evidence, we eliminate these observations, ensuring that the observations in the condensed sample are all subject to social media criticism. We thus use 5820 and 2014 observations to test CSR disclosure choice and substantiveness, respectively. We estimate Equations (1) and (2) and present the results in Table 8. Column (1) shows that in the probit regression where CSR is taken as the independent variable, CRITICISM still has a positive and significant coefficient (coefficient = 0.081, z-stat = 4.175). When RKS is added as the dependent variable, the results in Column (2) also show a positive and significant relationship, albeit slightly weaker, between social media criticism and the substantiveness of CSR disclosure. Most of the control variables are in line with our expectations. Overall, the robustness checks validate our main findings after changing the sample.

## 6. Further Analysis

As the need for CSR communication is different for companies with different attributes, CSR disclosure strategies in response to crises are expected to be heterogeneous. Property rights structure and government regulation lead to different legitimacy positions and corporate decision making on strategic CSR disclosure [41,59]. Therefore, the heterogeneous responses to social media criticism are influenced by company ownership and regulatory pressure. In this section, we analyze the heterogeneous effects of social media criticism posted by investors on the probability and substantiveness of CSR disclosure across SOEs and NSOEs and across regions with different levels of local government concern about environmental and social issues.

### 6.1. Effects on SOEs and NSOEs

Our empirical results indicate that SOEs are more likely than NSOEs to disclose CSR information and provide better CSR communication in reports. SOEs pay more attention to social responsibility because they are encouraged by the state to behave in socially and environmentally responsible ways [29,41]. This sub-section discusses the results of our investigation into whether SOEs and NSOEs behave differently with regard to CSR disclosure in response to social media criticism posted by investors.

To examine this issue, we partition the sample into SOEs and NSOEs and look at the effects of social media criticism on these two groups. As reported in Columns (1) and (2) of Table 9, the coefficient of CRITICISM is positive but statistically insignificant for the SOE group. However, CRITICISM has a significantly positive coefficient for the NSOEs group, and the difference between the coefficients for these groups is statistically significant. These observations suggest that a positive relationship between social media criticism and the probability of CSR disclosure only exists for NSOEs. Columns (3) and (4) report the estimation with RKS as the dependent variable. It also shows an insignificant relationship between CRITICISM and RKS for SOEs. The coefficient of CRITICISM is significantly positive and larger in the NSOE group than in the SOE group, confirming that NSOEs are more likely to issue substantive CSR reports in response to social media criticism.

These findings, although interesting, are not surprising. In emerging markets, political connections are an important form of capital, as business practices are heavily influenced by the government. Compared with SOEs, who enjoy innate legitimacy, Chinese NSOEs have less access to key resources due to their lack of government support, particularly during times of crisis. Social media criticism is therefore more likely to have negative effects on the organizational legitimacy and reputation capital of NSOEs, as SOEs operate with the endorsement of the government even if their reputation is partially damaged. To mitigate the adverse effects of social media criticism by investors, NSOEs are more likely to disclose their CSR activities and provide more substantive details on these actions to enhance their legitimacy. Our findings support the argument of Marquis and Qian [14] that compared with NSOEs, SOEs have greater legitimacy due to their inherent political ties and thus fewer incentives to engage in activities such as CSR reporting to seek access to resources.

### 6.2. Effects of Regulatory Pressure: Environmental Regulation and Social Concerns

In their CSR activities, Chinese companies pay the most attention to environmental protection. (According to Hurun China Social Entrepreneurship 2020, 36% of Chinese CSR activities focus on environmental protection, ahead of poverty alleviation, employee labor protection, community welfare and so on.) The reason for this is that, for a long time, China’s economic development has mainly taken place at the expense of its ecological environment, which has directly affected the health and well-being of its people. Other countries in the world have recognized and even condemned China’s serious environmental problems. Thus, the Chinese government regards environmental issues as the most important aspect of CSR and has launched a series of relevant actions to deal with them, such as revising “environmental law” and restricting lending to highly polluting enterprises or even closing them down. Guided by government, enterprises therefore devote much effort to environmental protection. Compared with the most prominent environmental issues, social issues such as human rights and employment conditions receive relatively little attention.) As documented in the literature [11], government pressure is one of the most salient factors affecting a company’s CSR behavior. We examine whether the effect of social media criticism posted by investors on strategic CSR disclosure is greater or smaller in regions with strict environmental regulations in place. We use local government spending on environmental protection scaled by budget expenditure each year to proxy for the intensity of local environmental regulation (REG). Our full sample is divided into two subgroups along the median of REG: companies in regions with high levels of environmental regulation (high-REG) and those based in regions with low levels of environmental regulation (low-REG). We then rerun the above models to account for these new subgroups. We observe in Columns (1) and (2) of Table 10 that the coefficient of CRITICISM is positive and statistically significant for the high-REG group and positive but insignificant for the low-REG subgroup. A test of the equality of the coefficients between the two groups implies that companies operating in regions where local governments place more emphasis on environmental protection are more likely to disclose CSR information in response to social media criticism. As indicated in Columns (3) and (4), the coefficient of CRITICISM is significantly positive only for the high-REG group, not for the low-REG group; however, the difference between the two groups is not statistically significant.

Although Chinese companies get most involved with environmental issues among the CSR activities, we cannot leave social issues unanalyzed, as they are important for improving people’s well-being and promoting social harmony and stability [21]. Therefore, we add social issues to the study and test whether the impact of investors’ social media criticism on CSR disclosure is greater or smaller in regions where local government is heavily concerned with social problems. We use the legal and institutional index (LI index) to proxy the level of local government concern for social issues. The LI index is one of five secondary indexes of the Marketization Index, which has been widely used in previous studies in the Chinese context [60,61,62]. The LI index evaluates the legal institutional environment in China’s regions from several angles, including the protection of social rights, effectively capturing the extent of government emphasis on social issues. We partition our sample into two subgroups based on the median of the LI index: companies in regions with high government concern for social issues (high-LI) and those in regions with low government concern for social issues (low-LI). We then rerun the above models. In Columns (1) and (2) of Table 11, we find that the coefficient of CRITICISM is positive and significant for the high-LI subgroup but negative and insignificant for the low-LI subgroup. As shown in Columns (3) and (4), the coefficient of CRITICISM is also significantly positive only in the high-LI group.

Overall, our results indicate that social media criticism has more obvious effects on CSR disclosure in regions where local governments attach greater importance to environmental and social issues. Given that enterprises in China are often dictated to by local governments, maintaining a good relationship with regulators gives companies better access to resources and support, which is critical to acquiring or maintaining a competitive advantage. Owing to the larger concern of increasing regulatory costs or sacrificing ties with local governments, listed companies in regions with higher levels of environmental regulation and social concerns tend to disclose CSR-related information and engage in more substantive CSR disclosures to showcase their commitment to social and environmental responsibility, as demanded by governments. Our findings also indicate that social media criticism from investors and regulatory pressure are effective in motivating companies to engage in CSR disclosure and substantive CSR behavior. These two external pressures—from investors and governments—work in tandem to achieve the same result.

## 7. Discussion

### 7.1. Practical Implications

First, this study has implications for the external stakeholders of listed companies in China. External stakeholders generally lack channels through which to voice their opinions on company operations, preventing them from playing a role in corporate governance. Studies have increasingly focused on the ways (e.g., conference calls, site visits) in which capital market participants can directly interact with companies or their managers [10,63]. However, this research has generally considered groups recognized as professional participants, such as institutional investors, analysts, and media representatives. Although non-professional groups (e.g., small shareholders, consumers, the public) are significant participants in the shaping of CSR actions, they have received little attention. We show that in the emerging market context, investor criticism posted on social media platforms can serve as a governance mechanism by prompting companies to release high-quality CSR disclosures. The widespread use of social media enables external stakeholders to play an effective role in corporate governance, publicly voicing their concerns to exert pressure on companies to reduce unethical and opportunistic behaviors.

Second, our findings have implications for policy makers in China. As corporations play a critical role in sustainable social and economic development, it is vital for policy makers to understand the factors that influence CSR. Our findings demonstrate that formal mechanisms such as environmental laws and regulations play a decisive role in CSR disclosure. Despite the growing popularity of CSR disclosure in China, many Chinese listed companies still provide only limited information on their CSR activities. Some companies even engage in “greenwashing” as a symbolic act. Our findings suggest that the growing number of crises linked to social media criticism increases the probability of a company engaging in voluntary CSR disclosure as well as substantive CSR reporting. In other words, social media criticism posted by investors can be an effective informal tool to influence the extent to which a company engages in CSR activities. Therefore, the Chinese government should strengthen its social responsibility governance. Regulators (e.g., the China Securities Regulatory Commission and stock exchanges) should advance the use of modern information technology in capital markets and provide direct channels for stakeholders to interact with managers to promote the sustainable development of listed companies. We find that the effects of social media criticism on CSR disclosure are more pronounced for NSOEs than for SOEs and for companies operating in regions with higher levels of environmental regulation and social concern. Therefore, multiple stakeholders should come together to guide CSR disclosure decisions.

### 7.2. Limitations

This study has several limitations that suggest directions for further investigation. First, we focus exclusively on companies’ CSR responses to criticism by investors on social media. While our findings fill an important gap in the literature, we acknowledge that different crises have diverse consequences for organizations, requiring a variety of crisis management strategies. We do not consider the effects of internal or external crises initiated by other stakeholders. For example, if a company is fined by regulators for financial fraud, how can it use CSR to mitigate potential risk? Second, as our main purpose is to investigate companies’ CSR decisions in response to crises, we do not empirically assess the outcomes of CSR disclosure. For example, do CSR disclosure choice and substantiveness minimize the corresponding adverse effects of social media criticism on organizational legitimacy? Third, we restrict our sample to public listed companies in China. We do not include unlisted private firms as they are not required to publicly disclose their CSR data and other financial data. Nevertheless, consideration of the CSR disclosure strategies of private firms is highly relevant to the line of research. Finally, we ask only whether the impact of social media criticism varies depending on ownership and regulatory pressure. We thus offer limited insights into heterogeneous CSR disclosure in response to crises. Do CSR disclosure decisions in times of crisis show different patterns across heavily polluted areas and other industrial sectors, or across companies with different political connections or board characteristics? Researchers should conduct comprehensive cross-sectional analyses to better understand the various attributes or situations that might affect a company’s CSR practices.

## 8. Conclusions

In this study, we investigate the ways in which Chinese companies respond to a crisis using CSR disclosure strategies. Differing from studies that have examined a series of factors that render a company more or less likely to disclose CSR activities, such as political pressure, we focus on how firms strategically use CSR communication in response to crises brought on by social media criticism by investors. Based on legitimacy theory and agency theory and using panel data on Chinese public companies listed on the SSE, we arrive at the following conclusions.

First, we find that companies tend to disclose CSR activities to gain legitimacy when they receive a large volume of social media criticism from investors. Although some studies have confirmed the importance of CSR and demonstrated that CSR engagement can build reputational capital for companies [33], few have discussed the ways in which companies use CSR communication as a tool for risk management in response to crises, especially those due to investors’ criticism. We focus on specific crises arising from negative comments by investors, a stakeholder group whose influence on CSR communication has not been adequately addressed in the literature [64]. Using the machine learning method, we identify criticism posted by investors via social media platforms and empirically test whether companies are more likely to voluntarily disclose CSR information when they are subjected to serious criticism on social media. Given that companies generally regard CSR disclosure as a legitimizing tool to communicate with investors and other participants, this practice can mitigate potential reputation damage resulting from social media criticism. Our results are consistent with those of Zhou et al. [16] and Ang et al. [27], confirming that investors play an effective monitoring role in corporate governance via their social media posts. This underscores the importance of investor criticism in forcing companies to avoid unethical behavior and engage in CSR disclosure.

Second, in addition to testing the propensity of companies to engage in CSR disclosure, which can be seen as a primarily symbolic act, we examine the extent to which CSR reporting reflects substantive CSR activities. CSR reporting in emerging economies has been criticized for its low quality and limited information content. However, these limitations have been neglected in studies of CSR disclosure in emerging markets. Using RKS rating data to evaluate the substantiveness of Chinese companies’ CSR reports, we find that criticism posted on social media by investors exerts pressure on companies to provide more substantive and detailed CSR reports. As stakeholders tend to criticize reports that do not provide substantial details of the ways in which companies intend to respond to investor criticism, listed companies are likely to engage actively in CSR rather than merely expressing a symbolic commitment to CSR. Communicating substantive details in CSR reports can help companies maintain their legitimacy by giving stakeholders insights into CSR initiatives in times of crisis. We expand research on CSR disclosure and show that crises can provide opportunities for companies to enhance their corporate image through disclosure.

Finally, our further analyses demonstrate that the effects of criticism on CSR disclosure are heterogeneous. Compared with SOEs, NSOEs are more likely to voluntarily disclose CSR activities in times of crisis. For SOEs, social media criticism does not significantly predict the probability of either CSR disclosure or substantive reporting. As NSOEs lack political connections, crises pose a considerable threat to their reputational capital and organizational legitimacy. NSOEs are thus more likely than SOEs to engage in crisis management through CSR disclosure. Furthermore, social media criticism only affects CSR actions in regions where the local government attaches importance to environmental and social issues. In other words, companies that operate in regions with stricter environmental regulations and high concern for social problems are more likely to engage in CSR activities and, crucially, to provide more substantive CSR reports in response to crises. Our findings reveal the heterogeneous nature of CSR responses to social media criticism and show how regulatory pressure from the government affects companies’ responses to investor criticism in terms of CSR disclosure and substantive engagement in CSR.

## Figures and Tables

**Table 1:** Sample selection and distribution.

Panel A: Sample Selection Process for CSR Disclosure Choice					No. of Observations		
Initial firm-year sample from 2013 to 2019					8703		
Less: Observations that are mandated to disclose CSR information					(2253)		
Observations with missing values for variables					(451)		
Final sample for testing disclosure choice					5999		
**Panel B: Sample Distribution for CSR Disclosure Choice by Fiscal Year**							
	2013	2014	2015	2016	2017	2018	2019
# of firm-years	612	622	679	776	1000	1094	1216
% of voluntary CSR disclosures	14.216	14.791	16.348	16.237	17.700	21.298	23.931
**Panel C: Sample Selection Process for CSR Disclosure Substantiveness**					**No. of Observations**		
Initial firm-year sample from 2013 to 2017					2249		
Less: Observations with missing values for variables					(150)		
Final sample for testing disclosure substantiveness					2099		
**Panel D: Sample Distribution for CSR Disclosure Substantiveness by Fiscal Year**							
		2013	2014	2015	2016	2017	
# of firm-years		384	393	412	438	472	
Average RKS score		41.261	43.312	42.761	43.507	43.023	

Note: This table presents our sample selection process for CSR disclosure choice (Panel A) and yearly distribution (Panel B), and the sample selection process for CSR disclosure substantiveness (Panel C) and yearly distribution (Panel D).

**Table 2:** Variable definitions.

Variables	Definition
Dependent variables	
CSR	Dummy variable that equals 1 if a firm voluntarily either issues a standalone CSR report or discloses CSR information in its annual report in year *t*, and otherwise 0
RKS	The natural logarithm of the overall rating score of CSR reporting substantiveness for a firm in year *t*, obtained from RKS
Independent variable	
CRITICISM	The natural logarithm of 1 plus the volume of criticism of a firm that investors post on the E-interactive platform in year *t*
Control variables	
SIZE	The natural logarithm of the book value of assets at the end of year *t*
LEV	The ratio of total debt divided by total assets at the end of year *t*
ROA	Return on assets ratio, defined as net income divided by total assets in year *t*
TOBINQ	The ratio of the market value to the book value of assets in year *t*
INSH	The sum of the percentage of stock holdings by institutional investors at the end of year *t*
FSH	The percentage of stock holdings by the first largest shareholder at the end of year *t*
OUTDIR	The proportion of independent directors on the board in year *t*
BOARD	The natural logarithm of the number of directors on the board in year *t*
SOE	Indicator variable that equals 1 if a firm’s ultimate shareholder is the state in year *t*, and otherwise 0
Industry	Dummy variable that represents industry (CSRC two-digit industry code)
Year	Dummy variable representing the year

**Table 3:** Sample descriptive statistics.

Variables	Obs.	Mean	Median	SD	Min	Max
CSR	5999	0.186	0.000	0.389	0.000	1.000
RKS	2099	3.711	3.670	0.297	3.054	4.413
CRITICISM	5999	2.816	2.890	1.175	0.000	5.298
SIZE	5999	22.185	22.089	1.254	19.452	27.955
LEV	5999	0.454	0.436	0.219	0.070	0.966
ROA	5999	0.033	0.034	0.066	−0.280	0.192
TOBINQ	5999	2.295	1.677	1.880	0.876	12.401
INSH	5999	0.386	0.398	0.235	0.000	0.921
FSH	5999	0.365	0.343	0.154	0.080	0.768
OUTDIR	5999	0.373	0.333	0.050	0.333	0.571
BOARD	5999	2.134	2.197	0.193	1.609	2.708
SOE	5999	0.429	0.000	0.495	0.000	1.000

Note: This table presents descriptive statistics for the main variables. The variables are defined in Table 2. All of the variables except the dummy variables are winsorized at the 1% and 99% levels.

**Table 4:** Pearson correlation matrices.

Variables	1	2	3	4	5	6	7	8	9	10	11	12
1-CSR	1											
2-RKS	−0.044	1										
3-CRITICISM	0.062 ***	0.025	1									
4-SIZE	0.258 ***	0.541 ***	0.151 ***	1								
5-LEV	0.074 ***	0.229 ***	−0.053 ***	0.413 ***	1							
6-ROA	−0.016	0.009	0.060 ***	0.010	−0.435 ***	1						
7-TOBINQ	−0.096 ***	−0.197 ***	−0.034 ***	−0.473 ***	−0.067 ***	−0.067 ***	1					
8-INSH	0.168 ***	0.340 ***	−0.042 ***	0.412 ***	0.184 ***	−0.013	0.003	1				
9-FSH	0.058 ***	0.191 ***	−0.083 ***	0.219 ***	−0.033 **	0.184 ***	−0.198 ***	0.279 ***	1			
10-OUTDIR	−0.004	0.064 ***	0.051 ***	−0.032 **	0.012	−0.029 **	0.076 ***	−0.060 ***	0.004	1		
11-BOARD	0.089 ***	0.240 ***	−0.072 ***	0.247 ***	0.127 ***	−0.028**	−0.125 ***	0.171 ***	0.037 ***	−0.560 ***	1	
12-SOE	0.140 ***	0.219 ***	−0.119 ***	0.254 ***	0.247 ***	−0.156 ***	−0.075 ***	0.377 ***	0.164 ***	−0.083 ***	0.246 ***	1

Note: This table presents Pearson correlation matrices of main variables. The variables are defined in Table 2. All variables except dummy variables are winsorized at the 1% and 99% levels. ** and *** indicate significance at the 5% and 1% levels, respectively.

**Table 5:** Test of H1: Social media criticism posted by investors and the probability of CSR disclosure.

Variables	Expected Sign	Dep. Var. = Prob (CSR)	
		(1)	(2)
		Coefficient	Marginal Effect
CRITICISM	+	0.066 ***	0.016
		(3.788)	
SIZE	+	0.202 ***	0.050
		(10.585)	
LEV	+	−0.423 ***	−0.104
		(−3.542)	
ROA	+	−1.057 ***	−0.261
		(−2.939)	
TOBINQ	?	−0.028 *	−0.007
		(−1.943)	
INSH	+	0.560 ***	0.138
		(5.652)	
FSH	?	−0.225	−0.056
		(−1.624)	
OUTDIR	+	0.852 *	0.210
		(1.837)	
BOARD	+	0.290 **	0.072
		(2.324)	
SOE	+	0.239 ***	0.059
		(5.414)	
Intercept	?	−6.376 ***	
		(−12.680)	
Year effect		Yes	
Industry effect		Yes	
Obs.		5999	
Pseudo R^2^		0.077	

Note: This table presents probit regression estimates for social media criticism and voluntary CSR disclosure choice. The variables are defined in Table 2. All of the variables except the dummy variables are winsorized at the 1% and 99% levels. The z-statistics are reported in brackets. *, **, and *** indicate significance at the 10%, 5%, and 1% levels, respectively.

**Table 6:** Test of H2: Social media criticism posted by investors and CSR disclosure substantiveness.

Variables	Expected Sign	Dep. Var. = RKS
		Coefficient
CRITICISM	+	0.011 **
		(2.389)
SIZE	+	0.081 ***
		(16.061)
LEV	+	−0.067
		(−1.632)
ROA	+	−0.046
		(−0.331)
TOBINQ	?	0.015 ***
		(2.651)
INSH	+	0.172 ***
		(5.874)
FSH	?	0.073 *
		(1.830)
OUTDIR	+	0.047
		(0.428)
BOARD	+	0.143 ***
		(4.872)
SOE	+	0.040 ***
		(2.967)
Intercept	?	1.271 ***
		(9.736)
Year effect		Yes
Industry effect		Yes
Obs.		2099
Adj R^2^		0.366

Note: This table presents OLS regression results for social media criticism and the substantiveness of CSR disclosure. The variables are defined in Table 2. All of the variables except the dummy variables are winsorized at the 1% and 99% levels. The t-statistics are reported in brackets. *, **, and *** indicate significance at the 10%, 5%, and 1% levels, respectively.

**Table 7:** Robustness check: Alternative proxy for CSR disclosure substantiveness.

Variables	Dep. Var. = RSK_Rank
	Coefficient
CRITICISM	0.017 **
	(2.368)
SIZE	0.121 ***
	(15.533)
LEV	−0.103
	(−1.538)
ROA	0.061
	(0.262)
TOBINQ	0.018 *
	(1.888)
INSH	0.279 ***
	(5.931)
FSH	0.071
	(1.131)
OUTDIR	0.002
	(0.009)
BOARD	0.204 ***
	(4.481)
SOE	0.049 **
	(2.183)
Intercept	−1.729 ***
	(−8.042)
Year effect	Yes
Industry effect	Yes
Obs.	2099
Pseudo R^2^	0.110

Note: This table reports the results of using an alternative measure of CSR disclosure substantiveness. The variables are defined in Table 2. All of the variables except the dummy variables are winsorized at the 1% and 99% levels. The z-statistics are reported in brackets. *, **, and *** indicate significance at the 10%, 5%, and 1% levels, respectively.

**Table 8:** Robustness check: Using condensed sample.

Variables	Dep. Var. = Prob (CSR)	Dep. Var. = RKS
	(1)	(2)
	Coefficient	Coefficient
CRITICISM	0.081 ***	0.009 *
	(4.175)	(1.701)
SIZE	0.201 ***	0.083 ***
	(10.346)	(16.031)
LEV	−0.396 ***	−0.087 **
	(−3.266)	(−2.081)
ROA	−1.045 ***	−0.099
	(−2.877)	(−0.698)
TOBINQ	−0.026 *	0.014 **
	(−1.793)	(2.493)
INSH	0.553 ***	0.177 ***
	(5.482)	(5.822)
FSH	−0.228	0.062
	(−1.621)	(1.496)
OUTDIR	0.856 *	0.023
	(1.818)	(0.205)
BOARD	0.265 **	0.148 ***
	(2.090)	(4.933)
SOE	0.239 ***	0.035 **
	(5.328)	(2.480)
Intercept	−6.358 ***	1.251 ***
	(−12.432)	(9.363)
Year effect	Yes	Yes
Industry effect	Yes	Yes
Obs.	5820	2014
Pseudo R^2^/Adj R^2^	0.076	0.370

Note: This table presents the regression results for the condensed sample. The variables are defined in Table 2. All of the variables except the dummy variables are winsorized at the 1% and 99% levels. The z-statistics/t-statistics are reported in brackets. *, **, and *** indicate significance at the 10%, 5%, and 1% levels, respectively.

**Table 9:** Differential effects on SOEs versus NSOEs.

Variables	Dep. Var. = Prob (CSR)		Dep. Var. = RKS	
	(1)	(2)	(3)	(4)
	SOEs	NSOEs	SOEs	NSOEs
CRITICISM	0.025	0.103 ***	0.000	0.024 ***
	(0.998)	(4.052)	(0.014)	(2.946)
		[*p*-value = 0.031]		[*p*-value = 0.007]
SIZE	0.227 ***	0.220 ***	0.088 ***	0.115 ***
	(6.851)	(7.915)	(13.793)	(10.401)
LEV	−0.805 ***	−0.143	−0.048	−0.231 ***
	(−4.658)	(−0.831)	(−1.038)	(−2.809)
ROA	−1.959 ***	−0.243	0.110	−0.716 ***
	(−3.582)	(−0.502)	(0.685)	(−2.665)
TOBINQ	−0.036	−0.016	0.015 **	0.032 ***
	(−1.440)	(−0.861)	(2.155)	(3.438)
INSH	0.565 ***	0.441 ***	0.202 ***	0.028
	(3.542)	(3.365)	(5.839)	(0.518)
FSH	0.168	−0.680 ***	0.016	0.209 ***
	(0.834)	(−3.420)	(0.334)	(2.692)
OUTDIR	0.931	0.215	0.067	−0.207
	(1.478)	(0.299)	(0.532)	(−0.860)
BOARD	0.429 **	−0.004	0.106 ***	0.255 ***
	(2.487)	(−0.020)	(3.210)	(4.036)
Intercept	−6.994 ***	−5.993 ***	1.435 ***	0.353
	(−9.264)	(−7.793)	(7.943)	(1.324)
Year effect	Yes	Yes	Yes	Yes
Industry effect	Yes	Yes	Yes	Yes
Obs.	2574	3425	1557	542
Pseudo R^2^/Adj R^2^	0.060	0.072	0.355	0.390

Note: This table reports heterogeneous effects for SOEs and non-SOEs. The variables are defined in Table 2. All of the variables except the dummy variables are winsorized at the 1% and 99% levels. The z-statistics/t-statistics are reported in brackets. The *p*-values for the differences between SOEs and NSOEs in the coefficients on CRITICISM are reported in square brackets. **, and *** indicate significance at the 5%, and 1% levels, respectively.

**Table 10:** Differential effects across regions with high and low levels of environmental regulation.

Variables	Dep. Var. = Prob (CSR)		Dep. Var. = RKS	
	(1)	(2)	(3)	(4)
	High-REG	Low-REG	High-REG	Low-REG
CRITICISM	0.097 ***	0.025	0.015 **	0.005
	(3.768)	(1.012)	(2.342)	(0.792)
		[*p*-value = 0.045]		[*p*-value = 0.281]
SIZE	0.225 ***	0.285 ***	0.085 ***	0.085 ***
	(8.523)	(8.353)	(12.403)	(9.925)
LEV	−0.522 ***	−0.572 ***	−0.044	−0.078
	(−3.065)	(−3.299)	(−0.763)	(−1.330)
ROA	−1.417 ***	−0.709	−0.102	0.214
	(−2.898)	(−1.331)	(−0.535)	(1.055)
TOBINQ	−0.019	−0.016	0.027 ***	0.003
	(−0.948)	(−0.768)	(3.364)	(0.417)
INSH	0.615 ***	0.285 *	0.126 ***	0.210 ***
	(4.471)	(1.914)	(3.222)	(4.666)
FSH	−0.258	−0.112	0.034	0.147 **
	(−1.339)	(−0.556)	(0.615)	(2.444)
OUTDIR	−0.261	1.855 ***	0.096	−0.031
	(−0.407)	(2.686)	(0.615)	(−0.195)
BOARD	0.246	0.211	0.086 **	0.219 ***
	(1.434)	(1.140)	(2.125)	(5.078)
SOE	0.243 ***	0.252 ***	0.047 **	0.044 **
	(3.985)	(3.843)	(2.310)	(2.332)
Intercept	−6.458 ***	−8.336 ***	1.242 ***	1.011 ***
	(−9.432)	(−9.989)	(7.161)	(4.597)
Year effect	Yes	Yes	Yes	Yes
Industry effect	Yes	Yes	Yes	Yes
Obs.	3069	2930	1059	1040
Pseudo R^2^/Adj R^2^	0.090	0.078	0.417	0.327

Note: This table presents the subgroup analysis for regions with different levels of environmental regulation. The variables are defined in Table 2. All of the variables (except the dummy variables) are winsorized at the 1% and 99% levels. The z-statistics/t-statistics are reported in brackets. The *p*-values for the differences between the high-REG and low-REG sub-samples in the coefficients on CRITICISM are reported in square brackets. *, **, and *** indicate significance at the 10%, 5%, and 1% levels, respectively.

**Table 11:** Differential effects across regions with high and low levels of social concern.

Variables	Dep. Var. = Prob (CSR)		Dep. Var. = RKS	
	(1)	(2)	(3)	(4)
	High-LI	Low-LI	High-LI	Low-LI
CRITICISM	0.119 ***	−0.019	0.012 *	0.004
	(4.577)	(−0.757)	(1.714)	(0.782)
		[*p*-value = 0.000]		[*p*-value = 0.137]
SIZE	0.283 ***	0.310 ***	0.082 ***	0.066 ***
	(8.571)	(9.117)	(10.385)	(8.615)
LEV	−0.225	−0.936 ***	0.090	−0.123 **
	(−1.264)	(−5.472)	(1.318)	(−2.506)
ROA	−0.500	−1.480 ***	0.165	0.075
	(−0.940)	(−3.045)	(0.729)	(0.456)
TOBINQ	−0.027	0.016	0.015 *	0.008
	(−1.211)	(0.789)	(1.680)	(1.083)
INSH	0.306 **	0.466 ***	0.168 ***	0.157 ***
	(2.247)	(2.903)	(3.914)	(4.045)
FSH	−0.317 *	−0.218	0.071	0.073
	(−1.651)	(−1.048)	(1.120)	(1.442)
OUTDIR	0.449	0.160	0.126	−0.062
	(0.647)	(0.244)	(0.726)	(−0.448)
BOARD	−0.416 **	0.787 ***	0.130 ***	0.174 ***
	(−2.126)	(4.626)	(2.767)	(4.731)
SOE	0.407 ***	0.088	0.041 *	0.053 ***
	(6.436)	(1.384)	(1.865)	(3.194)
Intercept	−6.826 ***	−9.301 ***	1.309 ***	1.575 ***
	(−8.431)	(−11.584)	(6.897)	(8.440)
Year effect	Yes	Yes	Yes	Yes
Industry effect	Yes	Yes	Yes	Yes
Obs.	3081	2918	1061	1038
Pseudo R^2^/Adj R^2^	0.107	0.089	0.427	0.273

Note: This table presents the subgroup analysis for regions with different levels of social concern. The variables are defined in Table 2. All of the variables (except the dummy variables) are winsorized at the 1% and 99% levels. The z-statistics/t-statistics are reported in brackets. The *p*-values for the differences between the high-LI and low-LI sub-samples in the coefficients on CRITICISM are reported in square brackets. *, **, and *** indicate significance at the 10%, 5%, and 1% levels, respectively.

## Data Availability

The data presented in this study are available on request from the corresponding author.
